# Mechanical Stress Is a Pro-Inflammatory Stimulus in the Gut: In Vitro, In Vivo and Ex Vivo Evidence

**DOI:** 10.1371/journal.pone.0106242

**Published:** 2014-09-02

**Authors:** You-Min Lin, Feng Li, Xuan-Zheng Shi

**Affiliations:** Division of Gastroenterology, Department of Internal Medicine, The University of Texas Medical Branch, Galveston, Texas, United States of America; Temple University School of Medicine, United States of America

## Abstract

**Aims:**

Inflammatory infiltrates and pro-inflammatory mediators are found increased in obstructive and functional bowel disorders, in which lumen distention is present. However, what caused the low level inflammation is not well known. We tested the hypothesis that lumen distention- associated mechanical stress may induce expression of specific inflammatory mediators in gut smooth muscle.

**Methods:**

Static mechanical stretch (18% elongation) was applied in vitro in primary culture of rat colonic circular smooth muscle cells (RCCSMCs) with a Flexercell FX-4000 Tension Plus System. Mechanical distention in vivo was induced in rats with an obstruction band placed in the distal colon.

**Results:**

In the primary culture of RCCSMCs, we found that static stretch significantly induced mRNA expression of iNOS, IL-6, and MCP-1 in 3 hours by 6.0(±1.4), 2.5(±0.5), and 2.2(±0.5) fold (n = 6∼8, p<0.05), respectively. However, gene expression of TNF-α, IL-1β, and IL-8 was not significantly affected by mechanical stretch. In the in vivo model of colon obstruction, we found that gene expression of iNOS, IL-6, and MCP-1 is also significantly increased in a time-dependent manner in the mechanically distended proximal segment, but not in the sham controls or distal segments. The conditioned medium from the muscle strips of the stretched proximal segment, but not the distal segment or control, significantly induced translocation and phosphorylation of NF-κB p65. This treatment further increased mRNA expression of inflammatory mediators in the naïve cells. However, treatment of the conditioned medium from the proximal segment with neutralizing antibody against rat IL-6 significantly attenuated the activation of NF-κB and gene expression of inflammatory mediators.

**Conclusions:**

Our studies demonstrate that mechanical stress induces gene expression of inflammatory mediators i.e. iNOS, IL-6, and MCP-1 in colonic SMC. Further ex vivo study showed that mechanical stress functions as a pro-inflammatory stimulus in the gut.

## Introduction

Inflammatory response in the gastrointestinal (GI) tract involves intricate coordination of numerous cellular and molecular events that are dictated by cytokines, chemokines, and other inflammatory mediators i.e. prostaglandins, nitric oxide and cell surface adhesion molecules [1], [2]. The inflammatory mediators may be produced by both inflammatory cells and non-inflammatory cells such as epithelial cells and smooth muscle cells (SMCs) in the gut [3]–[5], and have profound pathophysiological impacts on gut functions [1], [2], [6]–[9]. Prostaglandins and nitric oxide are well known mediators of gut motility function [8], [9]. Recent studies show that gut motility function is also markedly affected by cytokines such as IL-1β, TNF-α, IL-6, and intercellular adhesion molecule-1 [1], [2], [6], [10]. Furthermore, inflammatory mediators such as prostaglandins and cytokines also contribute to visceral hyperalgesia and abdominal pain [11], [12]. IL-6 is found to act on gut SMCs and sensory neurons, and affect both motility function and visceral sensitivity [10], [12]–[14].

Abnormalities in gut motility and visceral pain are well characterized pathological features in obstructive bowel disorders and some functional bowel disorders, in which lumen distension is present. Among these disorders are achalasia, chronic intestinal pseudo-obstruction, obstructive constipation, and idiopathic megacolon [15]–[19]. The pathogenic mechanisms of these functional abnormalities in these disorders are not well understood. Although it is commonly thought that no obvious gut inflammation is found in obstructive and functional bowel disorders, recent studies suggest that cytokines and pro-inflammatory mediators are increased systemically and locally in the gut in these conditions [20], [21]. The etiology of the increased cytokines and pro-inflammatory mediators in these conditions remains not well characterized. Moreover, inflammatory infiltration in the muscularis externae has been described in several functional obstructive bowel disorders such as chronic pseudo-obstruction [221, achalasia [23], and Hirschsprung's disease [24]. In chronic intestinal pseudo-obstruction, 30% of patients demonstrated inflammatory infiltrates (lymphocytes and mast cells) in the muscularis externae and myenteric ganglia [22]. Enterocolitis is a severe complication in Hirschsprung's disease, and the inflammation may not only be present in mucosa and submucosa, but also in the muscularis externae of the distended bowel [24]. However, the pathogenic mechanisms underlying inflammatory infiltrations in these conditions are not known.

The GI tract is consisted of a series of hollow organs, which are constantly subject to mechanical stimulations. Our previous studies found that lumen distention-associated mechanical stress markedly induced gene expression of COX-2 and subsequent increase of COX-2 derived prostaglandins (PG), i.e. PGE_2_ [25], [26] in gut SMCs. We found that COX-2, through its principal catalytic product PGE_2_, plays a critical role in motility dysfunction in bowel obstruction and other conditions with lumen distention [26], [27]. The so-called phospholipase A_2_/COX-2/prostaglandin E synthase /PGE_2_ (PCPP) axis is one of the best-studied pathways implicated in inflammatory regulation [28], [29]. We hypothesized that mechanical stress encountered in lumen distention may exert as a stimulus to induce expression of not only COX-2, but other pro-inflammatory mediators such as cytokines and chemokines in the gut wall. In the present study, we investigated whether mechanical stress induces gene expression of cytokines (i.e. TNF-α, IL-1β, and IL-6), chemokines (i.e. MCP-1 and IL-8), and other pro-inflammatory mediators (i.e., iNOS) in gut SMCs in the in vivo model of bowel obstruction and in vitro model of direct stretch in cultured colonic SMCs. We further determined if mechanical stress induced gene expression has pro-inflammatory actions in ex vivo experiments. Our study suggests that mechanical stress is a potent pro-inflammatory stimulus in the gut.

## Materials and Methods

### Ethics statement

The Institutional Animal Care and Use Committee at the University of Texas Medical Branch approved all procedures performed on the animals.

### Animal models of partial colon obstruction

Sprague-Dawley male rats weighing 200–275 g (Harlan Sprague Dawley, Indianapolis, IN) were used in the study. The rats were housed in a controlled environment (22oC, 12-hr light-dark cycle) and allowed food and water ad lib. The Institutional Animal Care and Use Committee at University of Texas Medical Branch approved all procedures performed on the animals.

The rat model of partial colon obstruction was prepared as previously described [25]–[27] with minor modifications. Rats were anesthetized with 2% isofluorane inhalation by an E-Z Anesthesia vaporizer (Palmer, PA). After midline laparotomy, a distal colon segment 4 cm proximal to the end of colon was carefully exposed. A small mesenteric window (5×5 mm^2^) was made next to the exposed colon segment. Partial colon obstruction was induced by placing a 3-mm wide medical grade silicon band around the colon wall through the small mesenteric window. The size of the silicon ring (21 mm in length) is 1–2 mm greater than the outer circumference of the colon when the colon segment is filled with fecal pellets, allowing a partial obstruction. The procedure to implement the silicon ring was completed within 2 min. The sham control rats underwent the same surgical procedure except that the ring was removed immediately after the 2-min procedure. Sham operated and obstructed rats were euthanized at different time points, 1 day, 3 days, and 7 days following operation. A 3-cm long colon segment starting at 1 cm oral to the site of obstruction was collected as stretched tissue, and a 2-cm-long colon segment starting at 0.5 cm aboral to obstruction was taken as non-stretched internal control. These tissues were used for biochemical and molecular studies.

### Tissue collection

The colon segments were collected in fresh carbogenated Kreb's buffer (in mmol/L: 118 NaCl, 4.7 KCl, 2.5 CaCl_2_, 1 NaH_2_PO_4_, 1.2 Mgcl_2_, 11 D-glucose, and 25 NaHCO_3_). The segments were cleansed, opened along the mesenteric border, and pinned flat in a petri dish with Sylgard base. The muscularis externae was separated from the mucosa and submucosa layers by micro-dissection as described previously [25]–[27], [30], [31].

### Primary culture of RCCSMC and in vitro mechanical stretch of SMCs in culture

Rat colonic circular SMCs (RCCSMCs) were isolated as described previously [25], [30], [32], [33]. In brief, the circular muscle tissue in 0.5×0.5 cm^2^ size was incubated in sterile HEPES buffer(in mmol/L: 120 NaCl, 2.6KH_2_SO_4_, 4KCl, 2CaCl, 0.6MgCl_2_, 25 HEPES, 14 glucose, and 2.1%essential amino acid mixture, PH 7.4) with 1.5 mg/ml collagenase (type II, 319 U/mg; Worthington, Freehold, NJ) and 1.0 mg/ml soybean trypsin inhibitor(Sigma-Aldrich) for 45 min at 31°C. At the end of digestion, tissue pieces were incubated in fresh buffer without digestion enzymes. The spontaneously dispersed cells were collected and cultured in DMEM supplemented with 10% fetal bovine serum (FBS) in the presence of 100 U/ml of penicillin G, 100 µg/ml streptomycin sulfate, and 0.25 µg/ml amphotericin B (invitrogen). The culture medium was changed every 3 days. Primary culture was allowed to grow for 8–10 days until cells were confluent. The cells were then seeded at 8×10^4^ cells/well in six-well BioFlex culture plates coated with type I collagen (Flexcell International, Hillsborough, NC). Cells were allowed to grow to ∼80% confluence before being subjected to DMEM/1% FBS for overnight prior to mechanical stretch [25], [30] via a FX-4000 Flexercell Tension Plus System (Flexcell International). This computer-regulated bioreactor applies multi-axial strain to cultured cells. Through vacuum pressure, cultured cells were deformed on flexible membrane plates. Cells incubated in parallel under identical conditions but without exposure to stretch served as controls.

### Culture of RCCSMCs in conditioned medium

The muscularis externae (200 mg) of the distended and control colon segments in the 3 day model were cultured in DMEM/1% FBS for 24 hours. The medium was harvested and taken as conditioned medium. The conditioned medium was applied to naïve RCCSMCs (in DMEM/1% FBS) [25],[30],[33] in 1∶2 dilutions to determine if inflammatory response is induced. In some studies, the conditioned medium was incubated with neutralizing antibody against rat IL-6 at 0.5 µg/mL or serum control for 1 hr before it was added into the cell culture of RCCSMCs.

### Immunofluorescence staining of NF-κB p65 in RCCSMCs

Immunofluorescent signal of NF-κB p65 in the primary culture of RCCSMCs was determined as previously described [4], [34]. Briefly, RCCSMCs were fixed in 1% paraformaldehyde in PBS for 30 min and permeabilized with 0.5% Triton X-100 for another 30 min. The cells were incubated with anti-NF-κB p65 subunit antibodies (1∶200 diluted in 3% normal goat serum) for 1 hour at 25°C. Rhodamine-labeled goat anti-mouse IgG (H+L) was used as secondary fluorescent antibody (1∶1000 dilution, 1 hour at 25°C). The cells were visualized under a Nikon microscope.

### Protein extraction and Western blotting

The muscularis externae tissue was homogenized on ice in lysis buffer supplemented with protease inhibitors (Sigma-Aldrich, St. Louis, MO). The compositions of lysis buffer are (in mmol/L) 20 Tris-HCl, pH 7.5, 150 NaCl, 1 EDTA, 1 ethylene glycol-bis (β-aninoethyl ether)-N,N,N',N'-tetraacetic acid, 2.5 sodium pyrophosphate, 1 β-glycerolphosphate, 1 Na_3_VO_4_, and 1% Triton X-100, and 1 ug/mL leupeptin. The proteins in the homogenates were resolved by a standard immunoblotting method as described previously (25–27, 30–34). Equal quantities of total protein (10 µg) were loaded and run on premade 4–12% Bis-Tris SDS-PAGE (Invitrogen, Carlsbad, CA). They were transferred to nitrocellulose membranes (BIO-RAD, Hercules, CA) for incubation with primary and secondary antibodies. The following antibodies were used in the study: primary antibodies to phospho-p65 (1∶200; Santa Cruz Biotechnology Inc, Santa Cruz, CA); β-actin (1∶5,000, Sigma, St. Louis, MO). Secondary antibody IRDye 800-conjugated anti-mouse IgG (Rockland, Gilbertsville, PA), or Alexa Fluor 680 goat anti-rabbit IgG (Invitrogen, Carlsbad, CA) were used. β-actin was used as loading control. The detection was done by Odyssey Infrared Imaging System (LI-COR Biosciences, Lincoln, NE).

### RNA preparation and real-time RT-PCR

Total RNA was extracted from tissues and cells by using the Qiagen RNeasy kit (Qiagen, Valencia, CA). One microgram of total RNA was reverse- transcribed by using the SuperScript III First-Strand Synthesis System (Invitrogen, Carlsbad, CA) for real-time RT-PCR (25–27). Real-time RT-PCR was performed with an Applied Biosystems 7000 real-time PCR system (Foster City, CA). For relative quantitation of gene transcription, real- time PCR was performed with 10 ng cDNA for the target genes and the endogenous control (L32) in a SYBR Green PCR master mix (Life Technologies, Grand Island, NY). PCR fluorescent signals of target genes were normalized to the fluorescent signal obtained by the housekeeping gene L32 for each sample. The primer sequences for the real-time RT-PCR assays are as follows: iNOS (sense: 5′-cggttcacagtcttggtgaaag-3′; antisense: 5′-acgcgggaagccatga-3′); IL-1β (sense: 5′-caccttcttttccttcatctttg-3′; antisense: 5′-gtcgttgcttgtctctccttgta-3′); IL-6 (sense: 5′-caaagccagagtccattcagagc-3′; antisense: 5′-ggtccttagccactccttctgt-3′); IL-8 (sense: 5′-ttggagacccctgcctgg-3′; antisense: 5′-acttctccacaaccctctgc-3′); TNF-α (sense: 5′-cccagaccctcacactcagat-3′; antisense: 5′-ttgtcccttgaagagaacctg-3′); MCP-1 (sense: 5′-tctcttcctccaccactatgca-3′; antisense: 5′-ggctgagacagcacgtggat-3′); L32 (sense: 5′-ttgctcacaatctgtcctctaagaa-3′; antisense: 5′-cgttgggattggtgactctga-3′).

### Multiplex immunoassay of cytokines and chemokines

Protein extraction for immunoassay of cytokines and chemokines was prepared as previously described [35]. The rat colonic muscularis externa was homogenized in cold PBS supplemented with protease inhibitors. LINCO rat cytokine/chemokine multiplex immunoassay kit (LINCO, St. Charles, MO) was used to quantitate cytokine/chemokine levels in the homogenates by following the manufacturer's protocols. The assay results were read and analyzed by a Bio-Rad BioPlex System powered by Luminex xMAP Technology (Bio-Rad Laboratories, Hercules, CA).

### Quantification of nitric oxide production

Colonic muscularis externae was isolated from the colon segment ∼2 cm oral to the site of obstruction. The muscularis externae pieces (3×10 mm) were incubated in 1 mL of phenol red free DMEM (Invitrogen, Carlsbad, CA) at 37°C for 24 hrs. After centrifugation at 500 g for 5 min, the tissue was weighed, and 40 µL of the supernatant was used for detection of nitric oxide metabolites using the nitrate/nitrite colorimetric assay kit (Cayman Chemical, Ann Arbor, MI). The nitrate/nitrite production was normalized by the tissue weight (nmol/g of tissue).

### Statistical analysis

All data points are expressed as means ±SEM. Statistical analysis was performed by analysis of variance with non-repeated measures (by Student-Newman-Keuls test) for multiple comparisons, and student's t-test for comparisons of two means. A *P* value of <0.05 was considered statistically significant.

## Results

### Mechanical stretch-induced gene expression of cytokines, chemokines, and iNOS in the primary culture of RCCSMCs in vitro

**Figure 1:**
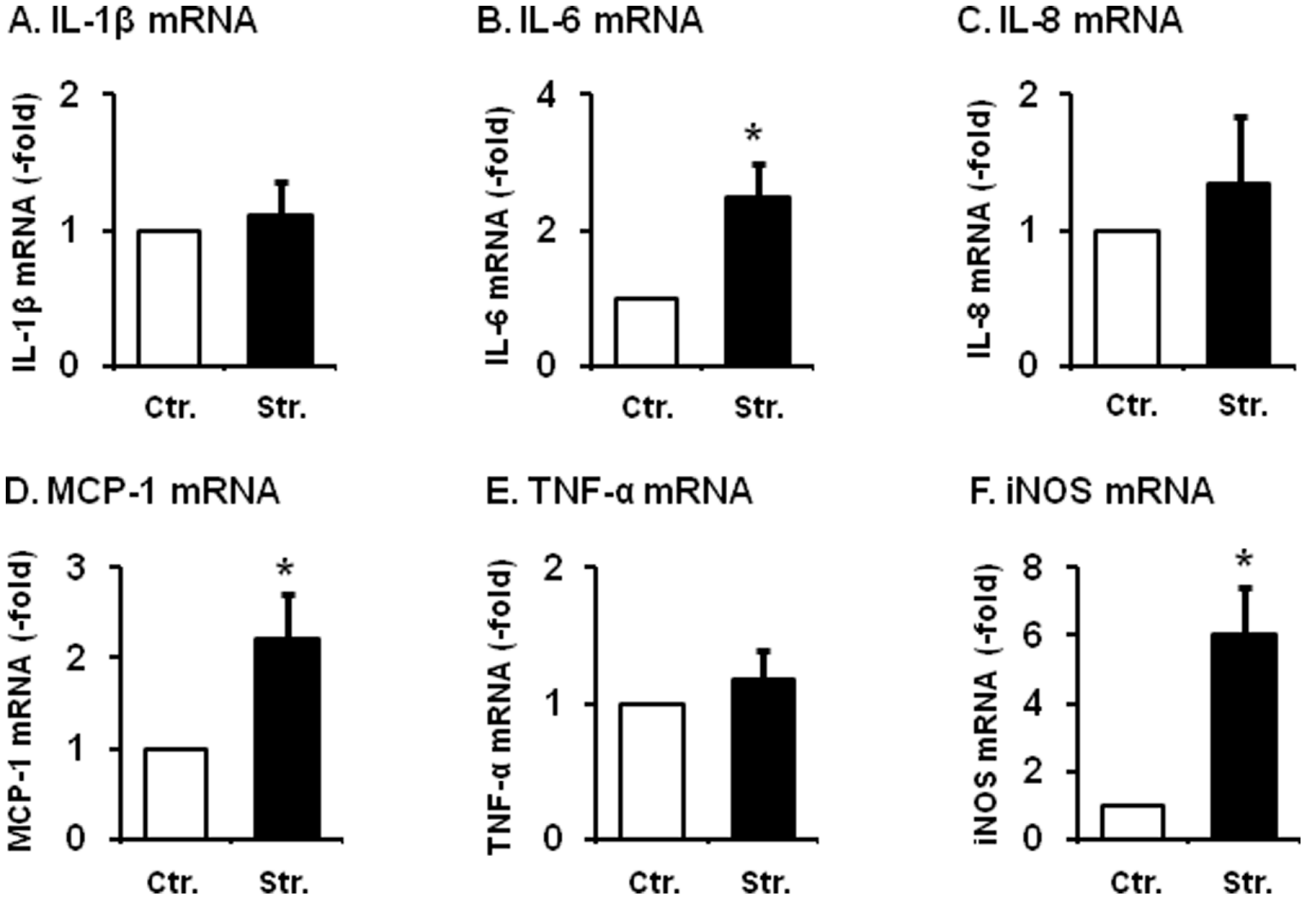
Mechanical stretch-induced mRNA expression of IL-1β, IL-6, IL-8, MCP-1, TNF-α, and iNOS in the primary culture of RCCSMCs. Cells were stretched statically with 18% length elongation for 60 min and harvested 3 h after the start of stretch. The controls (time point 0) were treated identically, except that they were not subjected to stretch. The iNOS mRNA increased by 6.0 (±1.4), IL-6 mRNA increased by 2.5 (±0.5), and MCP-1 mRNA increased by 2.2 (±0.5) fold, respectively (N = 6 to 8, p<0.05 in all). However, gene expression of TNF, IL-1, and IL-8 was not significantly altered by the mechanical stretch.

We first determined if direct mechanical stretch induced gene expression of cytokines, chemokines and proinflammatory mediators in vitro in the primary culture of rat colonic circular smooth muscle cells (RCCSMCs). Static stretch of the primary culture of RCCSMCs at 18% elongation for 60 min significantly increased mRNA expression of iNOS, IL-6, and MCP-1 (CCL2), but not TNF-α, IL-1β, and IL-8 (Fig. 1). Compared to the non-stretch control, the mRNA expression of iNOS, IL-6, and MCP-1 (CCL2) in the RCCMSC increased by 6.0(±1.4), 2.5(±0.5), and 2.2(±0.5) fold (n = 6∼8, p<0.05 in all), respectively at 3 hr after the start of mechanical stretch (Fig. 1).

### Gene expression of cytokines, chemokines and proinflammatory mediators in the model of colon obstruction in vivo

**Figure 2:**
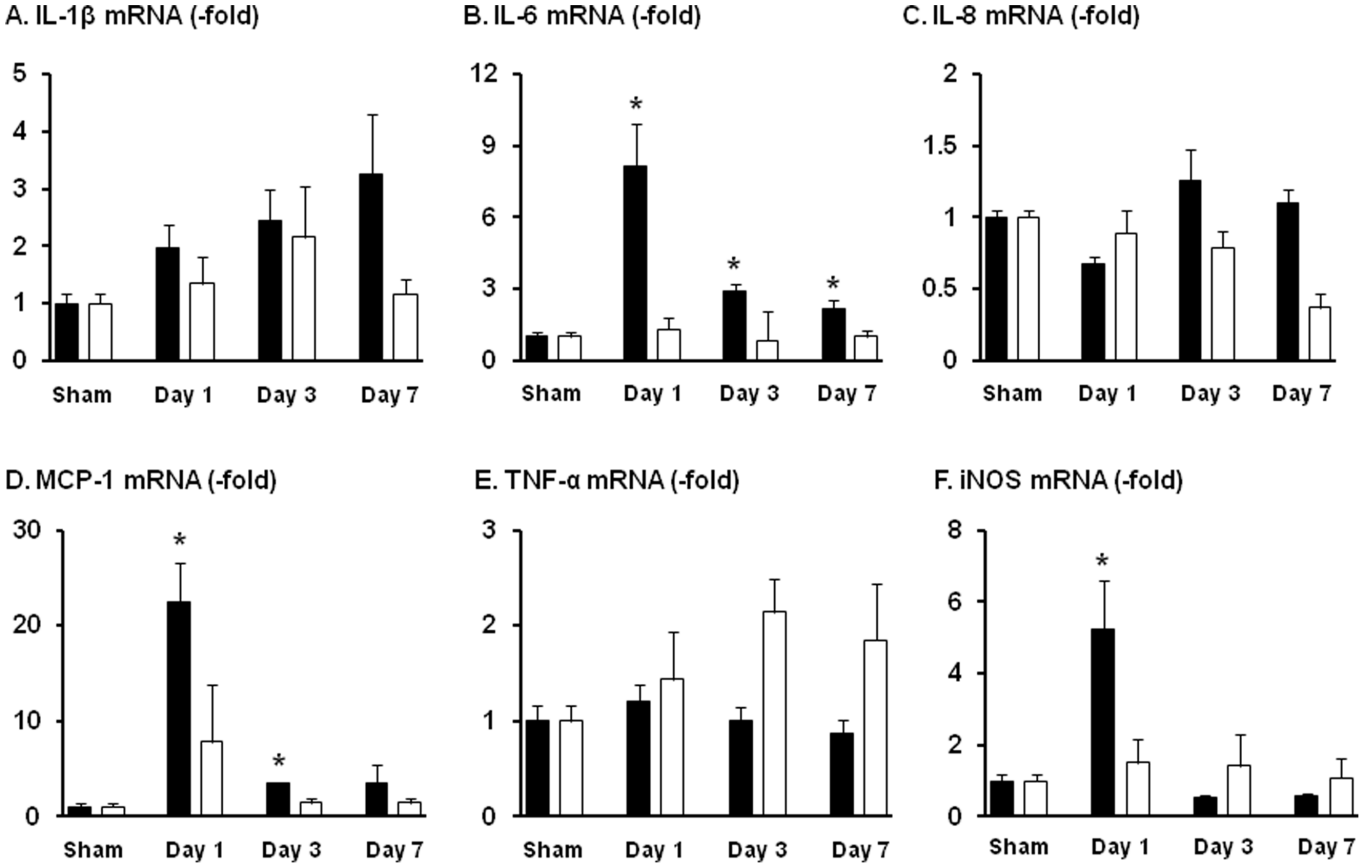
Lumen distention-induced mRNA expression of IL-1β, IL-6, IL-8, MCP-1, TNF-α, and iNOS in the colonic muscularis externae in rat model of partial colon obstruction. Rats were euthanized on day 1, 3, and 7 after the operation. Muscularis externae was isolated from the colonic segment oral (black bar) and aboral (white bar) to obstruction band for RNA extraction and quantitative PCR determinations. N = 8 rats for oral segment, and 4 for aboral segment. * p<0.05 compared to sham control.

In order to determine if mechanical stress induces up-regulation of proinflammatory mediators in gut smooth muscle in vivo, we compared the gene expression and secretion of cytokines, chemokines and proinflammatory mediators in a well established model of mechanical bowel obstruction [25], [26], [30]. In the rat model of partial colon obstruction, we found that the mRNA expression of iNOS, IL-6, and MCP-1 (CCL2) is significantly increased in the colonic muscularis externa of the mechanically distended segment oral to obstruction compared to sham controls (Fig. 2). The expression of IL-6 mRNA is increased at all time points (8.1±1.8 fold on day 1, 2.9±0.3 fold on day 3, and 2.2±0.4 fold on day 7; N = 6∼8 rats in each group; p<0.05 vs sham in all time points). The mRNA expression of MCP-1 (CCL2) was increased only on day 1 and day 3 (22.5±4.1 fold on day 1, 3.5±0.1 fold on day 3; N = 6∼8; p<0.05 vs sham control). The mRNA expression of iNOS was significantly increased in the segment oral to obstruction on day 1 (5.3±1.4 fold of sham control; N = 8; p<0.05 vs. sham). There was no significant increase of mRNA expression of these cytokines, chemokines and iNOS in the non-stretched distal colonic segment during the study period (day 1 to day 7) (Fig. 2).

**Figure 3:**
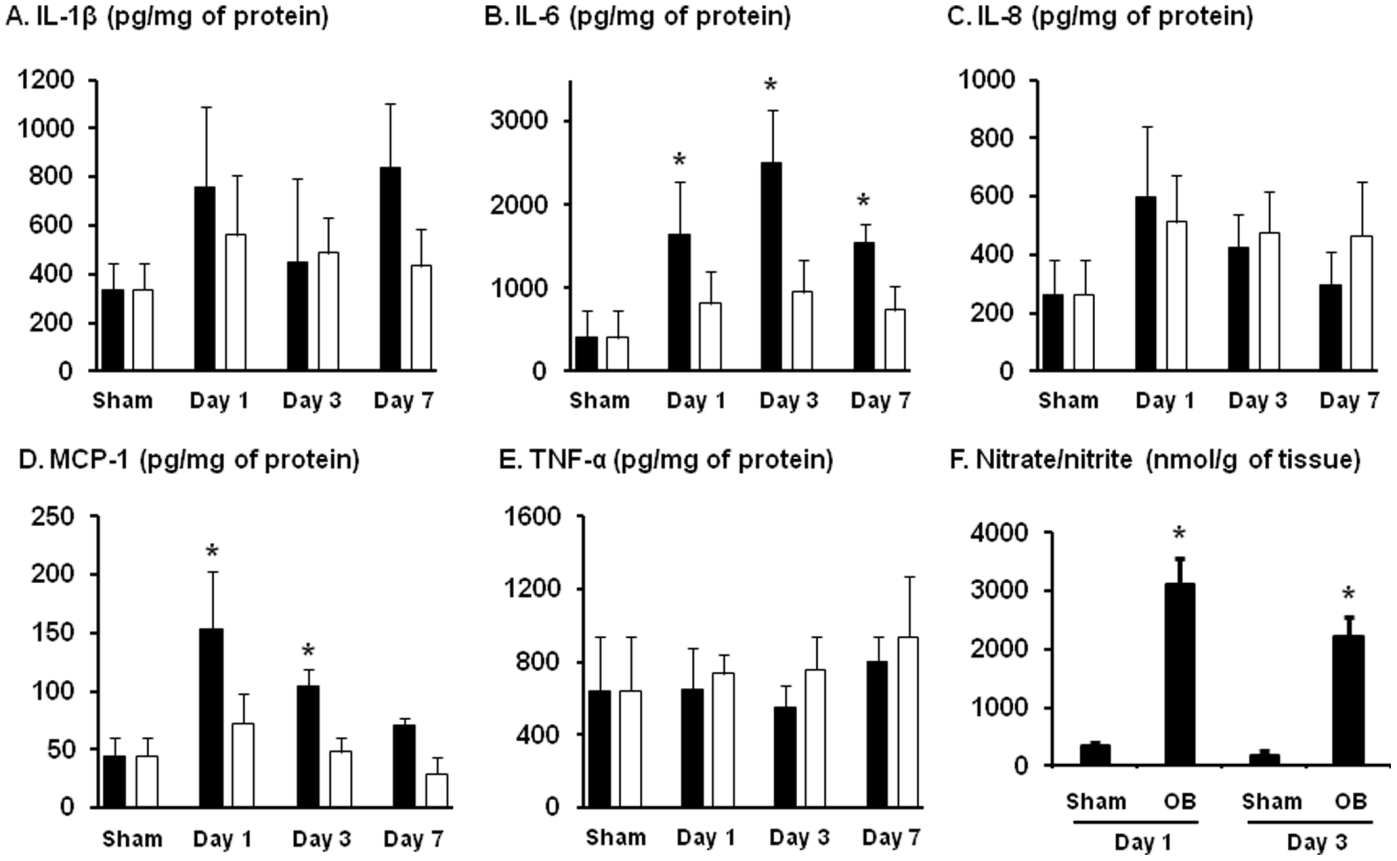
Detection of IL-1β (A), IL-6 (B), IL-8 (C), MCP-1 (D), TNF-α (E) and nitric oxide metabolites (F) in the colonic muscularis externae in rat model of partial colon obstruction. Rats were euthanized on day 1, 3, and 7 after the operation. Muscularis externae was isolated from the colonic segment oral (black bar) and aboral (white bar) to obstruction band for protein extraction and EIA quantification of cytokines and chemokines (A to E). Y-axis unit for each mediator from A to E is pg/mg of protein. The NO metabolites (nitrate/nitrite) from the colonic muscularis externae were detected in the incubation medium as described in the Methods (F). The Y-axis unit for F is nmol/g of tissue. N = 5 or 6 rats in each group. * p<0.05 compared to sham control.

Further LINCO cytokine/chemokine multiplex immunoassay confirmed that production of IL-6 and MCP-1 was significantly increased in the distended proximal segment, but not in the non-stretched distal segment (Fig. 3). The IL-6 production was significantly increased in all day1, day 3, and day 7. MCP-1 was significantly increased on day 1 and day 3. In addition, the mRNA and protein levels of IL-1β show a trend of increase in the later days of obstruction in the distended oral segment.

We also determined the production of nitric oxide (NO) in the muscularis externae of the colon segment ∼2 cm oral to obstruction site by measuring NO metabolites nitrate and nitrite (Fig. 3F). The nitrate/nitrite level was dramatically increased from 338.7±52.8 nmol/g in sham rats to 3121.4±438.1 nmol/g of tissue in the colonic muscularis externae of rats with obstruction for 1 day (p = 0.0002, n = 6). The nitrate/nitrite level (2227.2±338.4 nmol/g, p = 0.004) in obstruction decreased on day 3, but was still greater than in sham control (Fig. 3F).

### Activation of NF-κB in RCCSMCs by conditioned medium from mechanically distended colon

**Figure 4:**
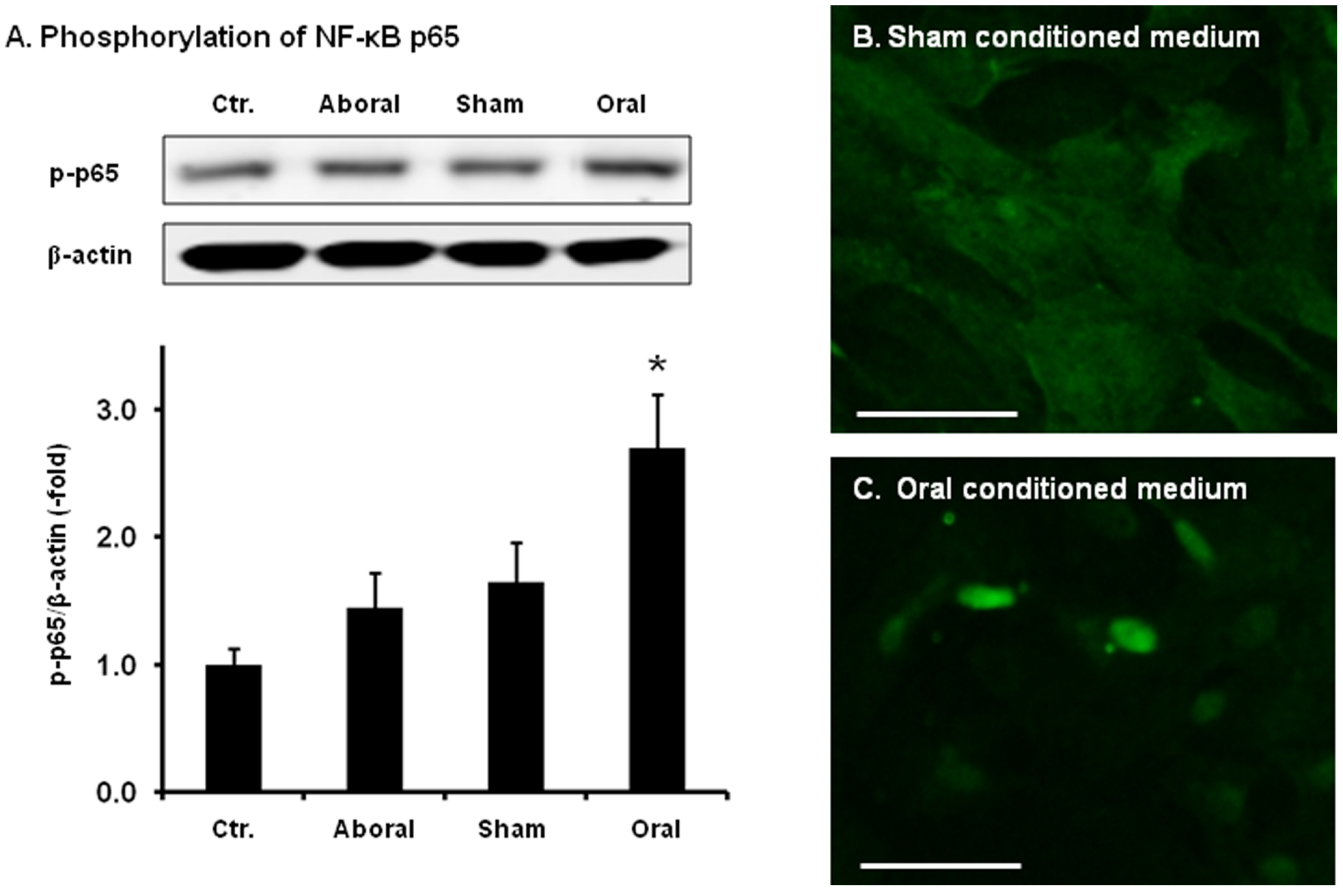
The phosphorylation and translocation of NF-κB p65 in RCCSMCS cultured with conditioned medium. (A) The NF-κB p65 phosphorylation was detected by Western blot with specific phosphor-p65 antibody. Cells were treated with conditioned media (1∶2 dilution) for 30 min. The p65 phosphorylation level increased 2.7±0.5 folds when naïve RCCSMCs were treated for 30 minutes with conditioned medium from the incubation of the oral segment (N = 4, * p<0.05 vs. blank control). (B) Immunofluorescence staining of p65 in RCCSMCs treated with conditioned medium from the sham tissue for 30 min. (C). Immunofluorescence staining of p65 in RCCSMCs treated with conditioned medium from the oral tissue for 30 min (Images shown are representative of 3 repetitions. Bars  = 100 µm.)

To determine if mechanical stress-induced gene expression has pro-inflammatory effects, we treated naïve primary culture of RCCSMCs with conditioned medium (1∶2 dilution) collected from incubations of colonic muscularis externa of the sham control, colonic segment oral to obstruction (oral) and colonic segment aboral to obstruction (aboral). Control medium and conditioned medium from the aboral colon had no detectable effect on activation of pro-inflammatory transcription factor NF-κB p65. However, the conditioned medium from the mechanically distended proximal colon (oral) significantly increased phosphorylation level of p65 (Fig. 4A). The p65 phosphorylation level increased 2.7±0.5 folds when naïve RCCSMCs were treated for 30 minutes with conditioned medium from the proximal segment (n = 4, p<0.05). Further immunofluorescence study found that the conditioned medium from the obstructed oral segment, but not the sham control, also markedly induced translocation of NF-κB p65 from the cytoplasm to the nucleus (Fig. 4 B and C, n = 3).

### Up-regulation of pro-inflammatory mediators in the RCCSMCs after treatment with conditioned medium from mechanically distended colon

**Figure 5:**
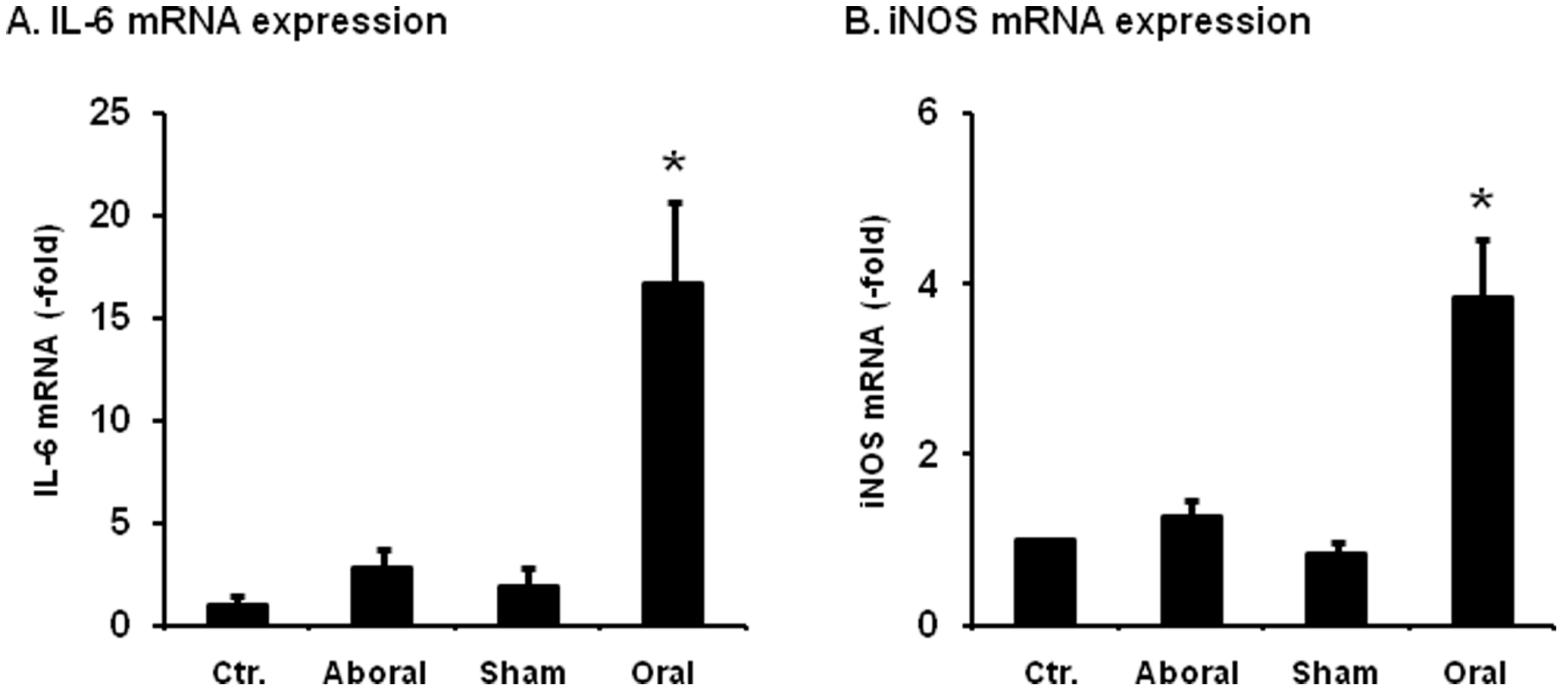
Expression of iNOS, IL-6, TNFα and IL-1β mRNAs in the RCCSMCS cultured with conditioned media. Cells were treated with conditioned media (1∶2 dilutions) for 3 hrs for RNA extraction and quantitative PCR determinations. N = 7 repetitions. * p<0.05 vs. sham controls.

We also determined if mechanical stress-initiated gene expression further induces NF-κB-dependent gene expression of inflammatory mediators (i.e. iNOS and IL-6) in the naïve RCCSMCs. Our data showed that the mRNA expression of iNOS and IL-6 in the naïve RCCSMCs was increased by 3.8 (±0.7) fold, and 16.7 (±3.9) fold, respectively after the treatment for 3 hrs with the conditioned medium from the mechanically distended oral segment (n = 7, p<0.05) (Fig. 5). However, conditioned media from the sham control and aboral segment did not have any significant effect on the mRNA expression of iNOS and IL-6 (Fig. 5).

### Effect of neutralizing antibody against IL-6 in the treatments with conditioned medium

**Figure 6:**
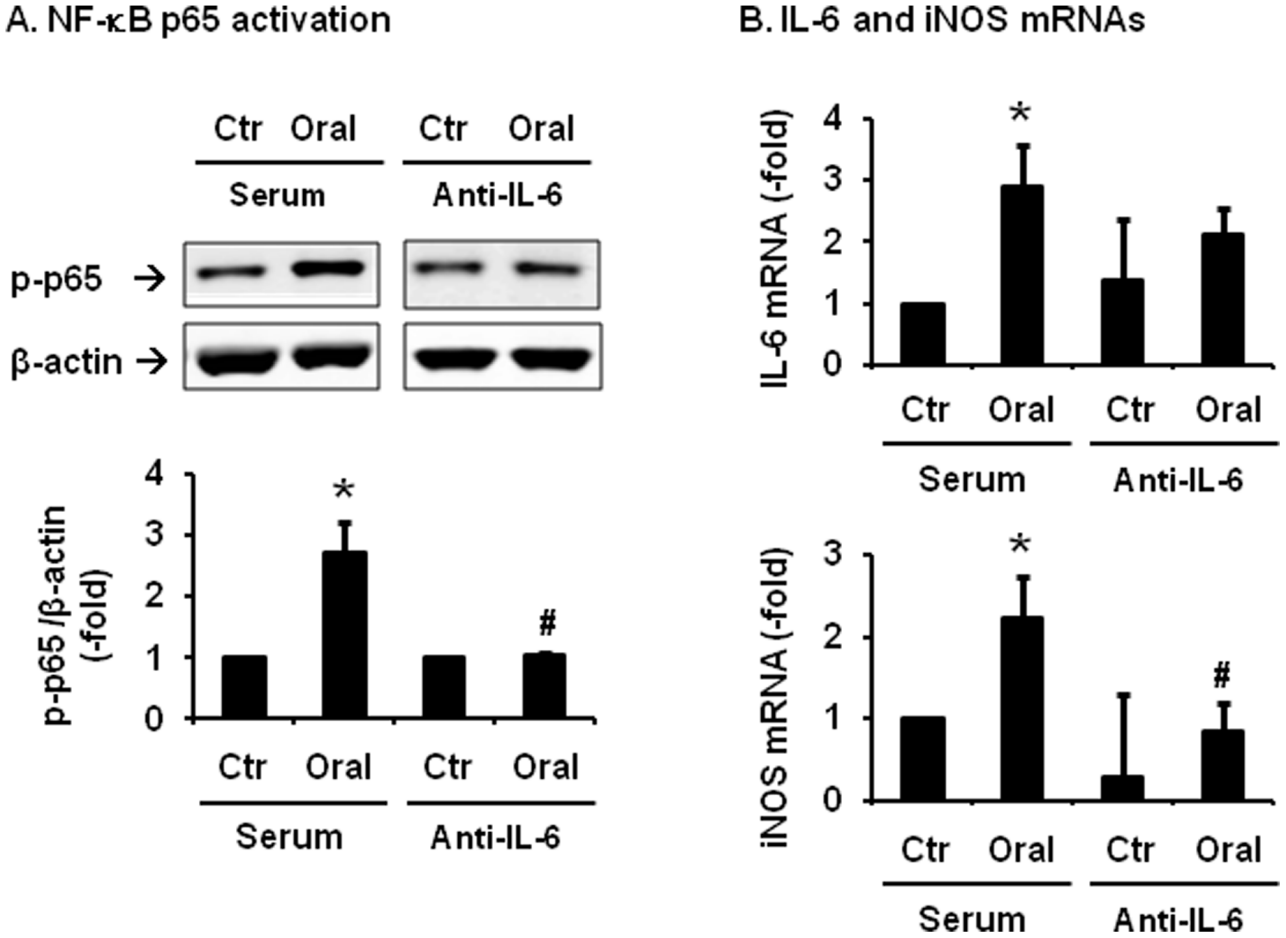
Effects of neutralizing antibody against IL-6 on activation of NF-κB and gene expression of pro-inflammatory mediators evoked by the conditioned medium. RCCSMCs were treated with control and conditioned medium from the obstructed colon (oral segment) in the presence of serum or neutralizing antibody against rat IL-6 (0.5 µg/mL). Cells were harvested in 30 min for the detection of NF-κB p65 phosphorylation (A) and in 3 hrs for the quantification of IL-6 and iNOS mRNAs (B). N = 5 independent experiments. *P<0.05 vs serum control. ^#^P<0.05 compared to the conditioned medium treated samples in serum group.

IL-6 is known to activate NF-κB and induce NF-κB-dependent gene expression of inflammatory mediators [36]. The expression of IL-6 was significantly induced in the distended colon in all the time points studied (day 1, day 3 and day 7) (Fig. 3). We then determined if treatment with neutralizing antibody against rat IL-6 (0.5 µg/mL) would attenuate activation of NF-κB and gene expression of inflammatory mediators induced by the conditioned medium of the oral segment. Our data showed that IL-6 antibody treatment significantly blocked the conditioned medium-induced activation of NF-κB and mRNA expression of iNOS (Fig. 6). The IL-6 mRNA expression increased 2.9(±0.6)-fold (p<0.05 vs. control) by the conditioned medium in the presence of control serum. However, this increase was blunted by the IL-6 antibody treatment (Fig. 6B).

## Discussion

In the GI tract, inflammatory mediators such as cytokines, chemokines, nitric oxide, lipid mediators, and cell surface adhesion molecules are produced not only by professional inflammatory cells, but also by non-inflammatory cells such as epithelial cells and SMCs [2]–[5]. Mounting evidence has shown that gut SMCs have synthetic function and may secrete cytokines, chemokines, and other pro-inflammatory mediators [4], [5], [7]. In all these studies, prototype pro-inflammatory cytokines (i.e. TNF-α or IL-1β) were used as stimuli [4], [5], [7] to test whether gut SMCs have the potentials to produce inflammatory mediators. In a cDNA micro-array study corroborated by real-time RT-PCR and EIA measurements, Shi and Sarna found that human colonic SMCs, upon stimulation of TNF-α, can produce a handful of cytokines, chemokines, and other inflammatory mediators such as IL-1β, IL-6, IL-11, IL-8, MCP-1, RANTES, and eotaxin [4]. The present study, however, demonstrates that mechanical stress is another potent stimulus in the gut to induce expression of cytokines, chemokines, and inflammatory mediators from the gut SMCs. We found in vitro that static mechanical stretch at 18% elongation induced gene expression of cytokines such as IL-6, chemokines such as MCP-1, and other pro-inflammatory mediators such as iNOS in the primary culture of RCCSMCs. We chose static mechanical stretch as in vitro stimulus because static stretch mimics well bowel obstruction and lumen distention in vivo [25], [26], [30]. Our previous studies found that static mechanical stretch also led to marked induction of COX-2 gene expression and increase of COX-2 –derived PGs in RCCSMCs [25], [26], [30]. A study by Wehner et al also found that static, but not cyclic stretch, induced expression of pro-inflammatory mediators such as COX-2, iNOS, and IL-1β in the primary culture of intestinal smooth muscle cells [37].

Furthermore, our in vivo study in animal model of lumen distention confirmed that mechanical stress is a potent stimulus to induce production of pro-inflammatory mediators. Interestingly, both our in vitro and in vivo studies showed that static mechanical stress induced expression of IL-6, MCP-1, and iNOS, but not TNF-α or IL-8. These data demonstrate that mechanical stress is a stimulus to induce selective expression of certain cytokines, chemokines, and pro-inflammatory mediators. Thus, the profile of pro-inflammatory mediators induced by mechanical stress in the colonic smooth muscle cells are distinctively different from that in inflammation [35] or that by inflammatory mediators such as TNF-α [4], [32]. In gut inflammation, almost all the tested cytokines and chemokines including IL-1β, TNF-α, IL-8 are increased in the muscularis externae [4], [32]. The study by Wehner using static stretch mode of 20% elongation detected significant increase of COX-2, iNOS, and IL-1β, but not IL-6. They did not confirm the expression profiles in any in vivo model of mechanical stretch. In addition, their study was on primary culture of small intestinal, but not colonic, smooth muscle cells [36]. It is possible that smooth muscle cells in the different organs may respond differently to mechanical stress [37]–[39].

Among the pro-inflammatory mediators inducible by mechanical stress, IL-6 and iNOS are well known to impair smooth muscle contractility in the gut [10], [40]–[42]. Increased IL-6 in the gut wall may also be involved in visceral hypersensitivity [43], [44] and chronic abdominal pain [45]. On the other hand, MCP-1 is a potent chemoattractant with an action of recruiting lymphocytes, monocytes and other inflammatory cells into the local site of the gut wall [2], [46], [47]. Our previous studies demonstrated that gene expression of COX-2 is markedly induced by mechanical stress in gut smooth muscle cells [25], [30]. COX-2 –derived PGs (i.e. PGE2), alike IL-6, not only exert potent effects on motility and visceral sensitivity, but also activate pro-inflammatory transcription factors [26], [28], [29]. We thus investigated in our ex vivo protocol whether mechanical stress-induced gene expression in gut smooth muscle cells is pro-inflammatory. It is found that conditioned medium of the mechanically distended colonic smooth muscle significantly activated NF-κB, and further induced expression of inflammatory mediators. Thus our studies demonstrate that mechanical stress is a potent pro-inflammatory stimulus in the gut. Further ex vivo studies demonstrate that stretch-induced IL-6 is a key mediator linking mechanical stress to its pro-inflammatory effects, as neutralizing antibody against IL-6 partially attenuated activation of NF-κB and up-regulation of pro-inflammatory mediators induced by the conditioned medium of the mechanically distended smooth muscle.

Several obstructive bowel disorders are considered “functional” or “idiopathic”, as the etiology and pathogenesis are not well defined. These disorders include achalasia, idiopathic chronic intestinal pseudo-obstruction, obstructive constipation, and idiopathic mega-colon. Motility dysfunction and chronic visceral pain are present in almost all these conditions. Given that lumen distention is commonly encountered in all these conditions, mechanical stress-induced gene expression of pro-inflammatory mediators i.e. COX-2, iNOS, and IL-6 may play a critical role in the pathophysiology of motility dysfunction and visceral pain in the disorders. Furthermore, studies have discovered that some of the “idiopathic” obstructive bowel disorders are associated with inflammatory infiltrates in the muscularis externae and myenteric plexus [22]–[24]. Based on our findings in the present study, it is logical to postulate that mechanical stress-induced chemokines such as MCP-1 may have a role in recruiting inflammatory infiltration in the disorders. Full-thickness biopsy of human tissue is warranted to detect if expression of cytokines and chemokines is altered in the muscularis externae of the enlarged bowel.

In summary, our studies in vitro and in vivo found that lumen distention and mechanical stretch up-regulate the expression of pro-inflammatory factors IL-6, MCP-1, iNOS, and COX-2 in the colonic smooth muscle cells. Mechanical stress-induced gene expression activates transcription factor NF-κB, and further induces production of pro-inflammatory molecules from the muscle cells. We conclude that mechanical stress is a potent pro-inflammatory stimulus in the gut.
